# Tuberculosis—Its Causes, and Indications for Its Treatment

**Published:** 1856-01

**Authors:** 


### ECLECTIC DEPARTMENT,

### AND SPIRIT OF THE MEDICAL PERIODICAL PRESS.

---

*Tuberculosis—its causes, and indications for its treatment. Inhalation.* By Henry Goadby, M. D., F. L. S.

Tuberculosis may fairly be considered as the opprobium of the medical profession. The want of information in regard to this disease has opened the door to the most extensive and varied forms of Quackery—which are at this instant of time more unblushingly impudent than probably at any former period. The object of this communication is to show that the phenomena of this disease is of an exceedingly simple and easily understood character. I first propose in the order of sequence to consider what constitutes tubercle. It must be obvious that this question can only be answered by reference to the Microscope. And it has fallen to my lot to examine the substance of "*tubercle*" microscopically on a great number of occasions. I have never seen but one uniform result, viz.: that tubercle consists entirely of the disintegrated and decomposed tissue in which it is found to be situated, whether Lung, Liver, or Intestine. In confirmation of this statement, that the matter of "*tubercle*" is made up of the debris of the tissue, it is only necessary to say that the field of the Microscope will be occupied with masses of the floating cell-walls, which still retain their capillary plexuses on both surfaces. So much, then, for what it really is. The next question is of the causes which have led to its production.

It invariably happens, in patients having a tendency to this disease, that the vital powers are depressed, and the circulation feeble. The heart, in fact, has not sufficient power to propel the blood throughout the capillary plexuses which eminently distinguish the tissues, the subjects of this disease. This remark especially applies to the pulmonary tissue, to which these observations will mainly refer.

In this organ, the capillaries are of less size, than any other such vessels in the human body, and complex and tortuous to the last degree. It is easy to conceive that any lack of contractile power in the heart, would fail to propel the blood through this wonderfully attenuated and difficult system of vessels. The circulation, therefore, fails to reach the ultimate capillaries throughout the pulmonary tissue, and if it fail for only a short space of time, what must be the inevitable result? Evidently, the circulation being once cut off, the part is left to die, and dying must succumb to the laws which regulate all dead matter; that is to say, it must be decomposed, and ultimately removed by absorption. In this case we are supposing that the powers of life, although depressed, are sufficiently good to carry on the process of absorption, which, however, is not true in by far the majority of cases.

Whenever a tubercle be established, either on the plural surface of the lung, or the interior of the tissue, nature appears to do all in her power to limit its extension; the colorless corpuscles of the blood are thrown out in great abundance, to form a line of demarcation, to separate the healthy structure from the rotting mass in its immediate vicinity. And if, in the meantime, the circulatory organs have acquired the necessary amount of energy, this process wiil surely be effected; but if not, the tubercle as necessarily extends. This, then, is one cause of tubercle; and another is to be found in the various causes which produce inflammatory action in the pulmonary tissue. In this latter, as in the former case, the primary mischief appears to be the cessation of circulation.

If we inflame a frog's foot under the Microscope, we shall see that the capillaries are instantly engorged, the blood beeomes intensely red, and the circulation entirely ceases. All due allowance should, of course, be made for the circumstances which attend inflaming, artificially, the *web of a healthy frog*, and inflammation which results from a positive depression, and disease. In the frog, simultaneously with the inflammation thus established, there is a sudden and remarkable aggregation of colorless corpuscles to the injured part. To them is delegated the function of repair. And through their agency, resolution of the inflammation soon results, and the blood resumes its wonte l channels. But, in the other case that we have supposed, the inflamed condition of the lung, the causes which have produced it, may continue, and set up such antagonism to the colorless corpuscles, as to defeat the intention of their exhibition. Moreover, the colorless blood-corpuscles may not co-exist with inflammatory action to the required extent. The too long continued arrest of the blood will render it impossible for the circulation ever to be resumed in the affected part, which is hence prepared to pass through all the phases which terminate in tubercle.

In the foregoing statements, we have supposed so slight a degree of tuberculosis, as every one is liable to, and the majority have experienced; for it is remarkable how few *thoroughly healthy lungs* I have been able to find in some hundreds of Post Mortem examinations. Neither is this a matter of any consequence, because we, all of us, possess much more lung than we have occasion to use, and can really afford to lose a great part of it with impunity.

There are cases in which the depression of the organs of circulation, already imagined, continues to exist. And the same depression precludes the possibility of absorption. In this case the *tubercle* remains, and becomes the source of further corruption by propagation.

There has been a lack of power to supply the colorless blood-corpuscles, to arrest the forming process of tubercle. Neither is it quite certain that the liquor sanguinis is provided with a sufficient quantity of these corpuscles, for this purpose, as it is obvious that depression of the organs of circulation, and impairment of the nutritive function, go hand-in-hand.

If healthy action can be established, the process of absorption becomes active, and the lungs heal by *cicatrix.* I have, in my Cabinet, a preparation of lung, from a woman 85 years of age, the pleural surface of which had been the seat of an infinity of cancerous tumors. These have cicatrized, and the cicatrices have become organized, a fact fully revealed by the Microscope, the preparation having been minutely injected.

The examination of tubercle, under the Microscope, exhibits, in addition to the debris of the structure of the tissue itself, *certain nucleated cells*, emphatically so called by "Paget." The question is, what are these cells, and how do they originate? Every Microscopist knows that it is very difficult to discriminate between a corpuscle of pus, a mucous corpuscle, and the colorless corpuscle of the blood. If my view of the nature of tubercle be correct, the first result is destruction of the tissue. From this moment, the tubercle goes through a variety of phases—in a downward direction—and may be favorable for all that I know to the contrary, to the ultimate production of corpuscles of pus; and I think likely that it really is. Hence their presence is accounted for. I have already indicated that the colorless blood corpuscles are thrown out in immense quantities to limit the spread of tubercle; and surely, it is no way wonderful that they should be found extensively associated with the matter of tubercle, although their presence as such, has been overlooked by authors.

Before hinting at treatment, plainly indicated by the above facts, it will be well to make a brief resume of the foregoing statement. Firstly, we find a depressed condition of the circulation, producing death in the tissue. Secondly, the tissue dies from the arrest of capillary circulation, the result of inflammation. Thirdly, the extension of tubercle by propagation. And, fourthly, the reparation by means of cicatrization.

Reasoning from these premises, the mode of treatment seems to be indicated as follows, viz: The power of *the heart must be increased*, and to effect this, we must have recourse to stimulants; and here we must inquire what stimulants should be considered the most judicious for this purpose? It should not be lost sight of, that energy and power should be given to the heart immediately. Every minute of delay is just so much valuable time lost. To meet such a contingency, we ask again, what is the best remedy within our present knowledge? and in my opinion, there is but one answer to this question, viz.: Brandy. I believe it should be administered in doses of from one to two tablespoonsfull, mixed with a little hot water, which greatly assists its action; and that this dose should be repeated at such intervals as circumstances may direct. I believe, however, that a far better remedy would be good old, and generous port wine; by which I do not mean the infusions of Logwood, too commonly sold for that fluid; *but the genuine wines of Oporto.* When this can be obtained I would prefer it, very much, to the brandy, the use of which is merely suggested as a substitute for the more appropriate fluid. Two or three wine-glasses per diem, of such wine as I have indicated, would prove of immense importance; because it would, of itself, supply nearly all that is requisite. The brandy contained in it, would sufficiently act upon the organs of circulation; whilst its nutrient principles would be fruitful in supplying the colorless blood corpuscle. The wine should be taken at intervals between meals; and every attempt should be made to form as large a quantity of fibrin, and albumen, in the liquor sanguinis as possible. To this end I would place the patient upon a light and nutritious diet, which should consist principally of any or all, of the many forms of Fecula, alternated with draughts of *beef-tea, so carefully prepared*, that a breakfast cup should contain the entire chemical elements of a pound of beef. To the latter I give the greatest importance, because it contains within itself, the very elements that are essentially requisite. The stomach may be in such a condition of debility that more than one ounce of solid meat, (if as much) would fail to be digested, and even then, at the expense of a long and difficult process. Whereas, if a larger quantity of beef be digested carefully in tho oven, and its elements dissolved out of it, the patient who cannot eat enough to sustain physical wants, can drink the concentrated essence of a pound of beef at a draught. Great care and attention is necessary in the preparation of beef tea, consisting of the two chemical elements already indicated: albumen, and fibrin. The boiling point should never, under any circumstances, be reached, for the very instant it be suffered to boil, the albumenous element becomes coagulated, and the fibrous element corrugated. And whenever this takes place, the process may be suspended, for no earthly power can extract further nutriment from the meat. In my experience the very best plan is the following: Take the lean of beef and hack, as if you were mincing it; put it into a clean sauce-pan, with just sufficient water to prevent it from burning; cover it up, and place it in an oven, so slow, that it cannot possibly boil; allow it to remain not less than from two to three hours, or longer if necessary, when a *light colored* fluid will be the result. Strain, and flavor it in any way most agreeable to the patient. The meat will be a mass of dry fibre.

I have been induced to give the rationale of beef-tea making, because I believe success depends upon the maintenance of scientific principles not generally clearly understood.

The plan of treatment here recommended, would seem to indicate a greater demand for the *cook* than the Doctor, but this is not really so. It requires the supervision and direction of a skillful Physician to regulate the treatment, to watch its effects, to ascertain the improvement of the pulse, to attend to the general secretions; and in fine, to give such further attention to the symptoms as the necessities of the case may require. Variations in the diet will frequently be rendered necessary; for example: When the stomach is supposed to be capable of it, a mutton chop, not too much cooked, might be substituted for a cup of *beef-tea.*

It will be seen by the facts adduced, that the processes of inhalation, so arrogantly put forth, at the present time, can have none other good than to fill the pockets of those who practice it. Of all the numerous class of pretenders, who affect to cure "Lung Diseases," this may justly be considered, as by far, the most *specious* humbug.

If this system of treatment have any effect at all, it must be of this kind: The air, passing through the bronchial tubes, and tubiales, ultimately reaches the air-cells. Suppose the inhaled material, possesses stimulating qualities, it may, (and is certainly supposed to,) act upon the capillaries. But what action does it produce there? If the circulation has been arrested, and the blood remains stagnant in the vessels, the utmost that can possibly result, will be the production of an oscillatory motion, such as is constantly seen in the frog's foot, which as it cannot lead to any practical result, will soon subside. Anything allied to renewed general circulation, by such agency, is altogether out of the question. The probability is, that so far from such effects taking place, the action is specifically upon the *air-passages*, and not the *air-cells.*

Inhalation would be far more likely to be active on a mucous surface than on a tissue, devoid of this membrane; which is the case with the *tubioles* and air-cells. As an expectorant and anodyne application, I can understand that inhalation may give relief—I only cannot believe that any permanent good is to be derived from this treatment as a cure for tuberculosis. It must be, in the nature of things, altogether inefficient; and whatever effects it is calculated to produce, are not likely to be permanent. But while the patient has been coquetting with this system, valuable time has been irrevocably lost; and that disease, which, in the first instance, would have succumbed to careful, legitimate and enlightened practice, may have made such inroad as to render recovery hopeless, if not impossible.

I would not be supposed to restrict the treatment to the plan which I have laid down, because other, and equally efficacious modes of building up the body, rescuing it from a state of prostration, are patent to the profession. Cod-liver oil, in certain cases, I believe to be a remedy of so much value, that we cannot sufficient'y extol its merits. Years ago, on the other side of the Atlantic, I have seen several apparently incurable cases restored by the use of this remedy.

Finally, I regard it highly important to sustain the heart's action, and employ a rigid and well-directed hygienic course of treatment.—*Peninsular Journal of Medicine.*

Detroit, Nov. 6th, 1855.

